# Interfaces of ‘being healthy and being Ill’: how is health being perceived by individuals with non-communicable chronic conditions?

**DOI:** 10.1186/s12939-024-02188-2

**Published:** 2024-05-27

**Authors:** Nilanjan Bhor, P Omkar Nadh

**Affiliations:** Indian Institute for Human Settlements, Bangalore (Bengaluru), India

**Keywords:** Determinants of health, Health system, Non-communicable chronic conditions, Perception of health, Urban health

## Abstract

**Background:**

Accommodating chronic care into the everyday lives of individuals diagnosed with non-communicable chronic conditions often poses significant challenges. Several studies in public health literature that addressed the question of non-adherence to treatment by turning their gaze towards individual’s perception of their own health restricted the use of perception exploration to visceral states and corporeality without adequately acknowledging the mutual permeance of socio-biological worlds. This study explored the socio-economic genealogies of individuals, to understand the role of structural and intermediate factors that determine health perceptions, by attempting to answer the question ‘how do individuals with non-communicable chronic conditions perceive their health as healthy or ill’?.

**Methods:**

This study was conducted in a low-income neighbourhood called Kadugondanahalli in India using qualitative research methods. A total of 20 in-depth interviews were conducted with individuals diagnosed with non-communicable chronic conditions. Individuals were recruited through purposive and snowball sampling.

**Results:**

The participants predominantly perceived their health as being healthy and ill in an episodic manner while adhering to their treatment and medications for chronic conditions. This was strongly determined by the factors such as presence of family support and caregiving, changes in work and occupation, changes in lifestyle, psychological stress from being diagnosed, and care-seeking practices. This episodic perception of illness led to the non-adherence of prescribed chronic care.

**Conclusions:**

Due to the episodic manner in which the participants experienced their illness, the paper recommends considering health and illness as two different entities while researching chronic conditions. It is important for the health system to understand and fix the healthy and ill episodes, which often lead to switching between controlled and uncontrolled states of diabetes and hypertension. To do so, it is important to consider the social, economic, behavioural and psychological factors in an individual’s health outcome. The interplay between these factors has socialized health perception and various related practices from the individual to the community level. Therefore, the health system needs to re-strategize its focus from individual to community level interventions to address the determinants of health and NCD risk factors by strengthening the NCD prevention approach.

## Introduction

Non-communicable diseases (NCDs), often considered as the diseases of the wealthy, are fast becoming prevalent among low-income groups in developing countries like India. The escalating burden of NCDs in India is evident from the recent estimates: 49% of all-cause mortality and 47% of all cause Disability Adjusted Life years reported in 2017 [24]. The need for chronic care in the everyday lives of individuals diagnosed with NCDs often pose significant challenges, particularly for those from vulnerable and disadvantaged socio-economic backgrounds. Policymakers have, more often than not, been misguided by the assumption that NCDs are diseases of the affluent and have not paid enough attention towards designing health systems keeping in mind low-income communities [17, 32]. As a result, the public healthcare system does not sufficiently accommodate the needs of low-income households while taking into account their socio-psychological factors.

The impact of NCDs, however, is not just about the health system. How should we understand the ways in which NCDs affect low-income communities? Beyond their epidemiological impacts, NCDs, like all illnesses, also have socio-cultural effects. This paper argues that socio-psychological factors shape and in turn get shaped by the individual perception of health in terms of ‘being healthy’ and ‘being ill’, which in turn determine care seeking practices. We will focus on how this occurs specifically with NCDs in low-income communities.

Policymakers have rightly identified that care seeking practices and non-adherence to treatment are significant reasons for negative health outcomes among individuals diagnosed with NCDs in low-income communities [4]. However, only the addition of NCD treatment to existing health systems, which is a current policy practice, does not sufficiently address this problem. Several studies in public health literature have now begun to address this question of non-adherence to treatment by turning their gaze towards individual’s perceptions of their own health [14, 15, 25]. Nevertheless, these studies restrict their use of perception exploration to visceral states and corporeality without adequately acknowledging the mutual permeance of socio-biological worlds, i.e. considering the individual perceptions of health and illness in relation to the social environment. Such explorations often overlook the broader structural factors that play a determining role in shaping health perceptions. Therefore, this study attempts to move beyond this approach and understand perceptions of health in terms of ‘being healthy and being ill’ by taking into account the symbiotic socio-biological relationship involved. Following Das et al. ’s [9] idea on the notions of being healthy and being ill, where “the diseased state of an individual does not inevitably lead to ill-health and that absence of diseased state is also not an assurance for good-health”, we argue that an individual’s perception of health is shaped not just by physiological factors but also by a multitude of socio-psychological factors. We also further argue that the individual’s perceptions of their health plays a significant role in shaping their socio-economic conditions.

The study examines the socio-economic genealogies of the participants to understand the role of structural factors in determining perceptions of their own health and its attendant practices. It considers that ‘being healthy and being ill’ is not just shaped by the physiological/biological factors, but also social, economic and psychological factors, play an important role in shaping diagnosis, adherence to treatment and the very notion if one is ill. Additionally, the study addresses questions related to how the onset of chronic conditions reinforces the already existing structural inequalities in terms of diminishing degrees of freedom (biologically, economically, socially) and thereby life chances- ability to lead a decent life. Particularly, in the context of this study, our findings reveal that perspectives on being healthy and being ill are discursively articulated by individuals as relating to their family support, work and occupation challenges, lifestyles, prior knowledge of the condition and care seeking practices, all of which are significantly tied to the underlying social structures. Identifying and understanding the range of factors that shape the full care seeking cycle, from recognition and diagnosis to adherence to treatment and care seeking, is important to understand public health.

## Methods

The study takes an anthropological approach to understand the perceptions of health through the categories of ‘being healthy and being ill’ in a low-income neighbourhood. For this purpose, KG Halli, one of the 198 administrative units of the city of Bangalore, was selected. KG Halli has a population of more than 34,842 individuals spread over 0.7 square kilometres [3]. It is largely a low-income settlement with a recognised slum. A majority of people follow Islam (60%) followed by marginalised caste Hindu groups. A significant number of intergenerational, interstate migrants also reside in the neighbourhood. As the socio-demographic data is not publicly available, we made conclusions based on our interaction with the residents over 1.5 years [3].

The participants, who consented to the interview, fell under three categories related to diagnosis of hypertension and diabetes: (i) self-reported and diagnosed: this refers to a chronic condition that has been self-reported by individuals and has also been confirmed by a health service provider (ii) diagnosed and currently receiving treatment: this refers to individuals are receiving some form of treatment (including medications) whether diagnosed or self-prescribed (iii) diagnosed without treatment (this refers to individuals who are diagnosed but are receiving no form of treatment). In this study, we adhere to the guidelines of the Consolidated criteria for reporting qualitative research (COREQ) checklist [33].

Data was collected from January 2020 to April 2021 using open ended in-depth interviews. A total of 20 in-depth interviews were conducted face-to-face with individuals diagnosed with either diabetes and/or hypertension who were recruited using a purposive and snowball sampling method. The number of interviews were decided based on the saturation levels reached during the course of conducting interviews. Care has been taken to ensure that the sample represents the socio-economic diversity of the neighbourhood. Interviews were conducted by both the researchers (authors). in Hindi, Kannada, Telugu and English languages. For Tamil language interviews, translation help from our additional project staff was sought. Participants (65% female and the rest male) of aged 40 years and above from different religious, caste and income backgrounds were interviewed in their own houses in the language of their choice (Hindi, Tamil, Kannada, Telugu, English-1 participant) with the help of an open-ended interview guide that covered aspects of their intergenerational socio-economic conditions, education and occupation details, prior knowledge of the conditions of hypertension and diabetes, treatment and care seeking practices. The interview guide was piloted and then implemented for data collection. The final interview guide was also improved based on information that emerged in the course of data collection. Consent was taken from participants to audio-record the interviews. Field notes were also documented systematically during each interview based on the themes of inquiry and analysed likewise. The average duration of each interview was about 30 min.

The recordings of these interviews were transcribed in the same language the interviews were conducted (i.e. in Hindi, English, Kannada, Tamil and Telugu) and then translated into English. Interviews conducted in English were directly transcribed into English (Only one female participant). Next, multiple readings of the transcripts were performed to generate codes and recognise the formal themes by using ATLAS.ti9 software. Data was broadly segregated and organised under various themes reflecting the participants’ intergenerational socio-economic status, their prior knowledge of the conditions, treatment and care seeking practices, the occupational burden of the chronic conditions, and family support and care giving. Each of these sub-themes were found to influence the perceptions of diabetes and hypertension among the participants. Data coding and analysis were performed by the researchers based on the thematic segregation. We examined the elements based on the extent of their recurrence and centrality to understand the relationships that determine the perceptions of being healthy or being ill by the participants.

Institutional ethics approval was obtained from Indian Institute for Human Settlements to conduct the research at KG Halli. Further informed verbal consent was obtained from each participant, who have given their autonomous decision on willingness to participate, before commencement of each interview and, at the same time, the process also entailed explaining the study information such as purpose and objectives of the research, confidentiality aspects, and rights to participate and withdraw from the interview. Verbal consent was documented using audio recorder where participant’s identity i.e., the names of the participants were replaced with unique identity numbers, and hence, participants names were kept confidential during the transcription and translation activities. Due to confidentiality, the study participant’s names are also not being used in this paper, and represented as ‘P’.

## Findings

The study participants—who were diagnosed with either diabetes or hypertension or both—articulated their perceptions of health through the notions of being healthy and being ill. Our findings reveal that the study participants predominantly perceive being healthy or being ill in an episodic manner while adhering to treatment and medications for diabetes and hypertension. This episodic nature of being healthy and being ill leads to the non-adherence of the prescribed chronic care to manage NCDs. In this paper, we show that this perception of being healthy sometimes and being ill at other times that in turn determines care-seeking practices is influenced not only by the physiological imbalances through the controlled and uncontrolled state of diabetes and hypertension, but also by social, economic, behavioural, and psychological factors. The paper also delineates the role of perceptions in shaping these factors. Each of these factors and their relationships with individual perceptions of health are discussed in the following sections.

### Health perceptions during diagnosis and management of NCDs

The perception of being healthy or ill is articulated by the participants before the diagnosis and during care-seeking. Before the diagnosis, “*takleef*” (physical discomfort), “stress” and “tension” were used to express being ill, which the participants have connected with their diagnosis of either diabetes and/or hypertension.

While the perception of being ill is strongly connected to physical discomfort among the participants, we observed that our participants typically consider this illness to be episodic. They initially normalised their physical discomfort and did not seek early care because of their lack of awareness that their symptoms could be of underlying chronic conditions like diabetes and hypertension. Our findings also reveal that the local healthcare providers too often overlooked this connection. As an immediate remedy to their episodic physical discomfort, an injection or symptomatic tablet that gives quick relief was prescribed. Therefore, care-seeking by the participants is affected by the combined effort of their perception of being healthy and the non-recognition of their symptoms by the healthcare providers. This in turn delays the early diagnosis and initiation of the treatment of diabetes and hypertension. Our observations revealed that the duration of this delay varies based on the economic conditions of the individuals and perception of their own health.

It used to hurt my knees and I used to face a lot of difficulty in sitting, standing up, walking and turning around. Even when I had to walk long distances, I used to sit on a big stone, take some rest in between and then continue walking. This has been the case for the last 5–6 years. Taking injections or medicine gives some relief for the time being but the problem persists P(3).

However, the majority of the participants with the continuous episodic recurrences have been diagnosed in much later stages from the onset of the symptoms. P(1) articulated as,

I didn’t even know that I had diabetes initially. I had BP and I used to take injections and go for check-ups. Then my knees used to hurt and I used to go take injections twice, thrice a week. Later I went to a public hospital for a check-up and I was diagnosed with diabetes.

As is evident from the above quotes, participant’s inability to distinguish the episodic physical discomfort as a symptom, from a normalized pain in everyday life resulted in the lack of diagnosis of diabetes and/or hypertension and when to seek care.

In addition, stress due to livelihood struggles, familial problems and life events such as deaths or other illnesses among family members, were also commonly stated by the participants as shaping their perceptions of illness. One participant, whose brother was suffering from mental illness, said

He [participant’s brother] was mentally affected. He was drinking and smoking also. He was taking tablets from NIMHANS hospital. With the tablets he did all these things so he was mentally affected. So we shifted him to a hospital. I’m the younger sister. We were tense. At that time, moving here and there, with the tension I got it [blood pressure] P(8).

Participants also used terms like *taqdeer* (fate) to describe their social and economic circumstances and their psychological problems. P(5), who lost their spouse and considered losing spouse as fate, articulated this as,

It’s my fate. After my husband’s death, my elder daughter came to me with her girl child. Looking at her [daughter] condition, I used to get stressed and that gave me diabetes, BP and thyroid problems.

Further, the diagnosis of diabetes and/or hypertension itself led participants perceive their health as being ill. We understood from the interviews that recurring episodic illnesses led local health practitioners who were immediate care providers in the neighbourhood to recommend diagnostic tests. P(4) articulated “ *after I was told [by the doctor] I had it [BP and sugar], I automatically began feeling weak and I began wondering, why did it happen to me? … I have been weak since then*.”

Management of the chronic conditions as an everyday exercise also influenced in participants perceiving their health as being ill that is not associated with actual physical discomfort

P(2) said, “*This [managing medicines] keeps reminding me every morning, evening and night. Whenever I have to take the tablet I am reminded of it that I am ill. If in any way taking the tablet stops, then this memory will go away, the thought itself will go away”*.

This influenced perception of being ill sometimes acts against continuing regular care and adhering to the “course” of medicines. According to P(10), medicines are not required if BP/sugar level is under control, “*If BP measure is normal there is no need to take medicine. If BP goes out of control, then I take medicine*.” Another participant P(8) intentionally did not consume her routine tablets, said that “*I deliberately keep missing my BP tablet for a few days. I miss it, questioning the need to take so many medicines.*” This irregular intake of medicines due to the perception of being healthy is likely one of the main reasons for diabetes and hypertension being uncontrolled.

However, all the participants had medicines stored in a typical box as a response to their episodic symptoms as stated during the interviews. For instance, P(3) stated, “*I keep a stock of medicines. There is paracetamol and calcium tablets [including BP and Sugar tablets]. If I have slight pain or fever or cold then I consume medicines.*” Symptomatic medicines are sought as a quick relief from physical discomfort and other short-term morbidities.

Few participants also recognised the importance of regular intake of medicines for diabetes and/or hypertension due to persisting physical discomfort that most often than not generates a feeling of illness. P(12) articulated

“*Every morning I have to consume these two tablets for certain or else I have pain in my knees and my back. Nothing makes sense to me when I am in pain. Doctor advised that I don’t miss my medication so I take them regularly”*.

### Perceiving health through family support and caregiving

Family support and caregiving were social factors that strongly influenced the perception of being healthy or being ill. The support and care-giving reflected through the presence or absence of active support, from family members, especially children (mostly married children). P(12) articulated this as “*I spend my days very peacefully without any tension. My daughter-in-law doesn’t let me do any work and takes good care of me. My son also takes good care of me*”. Participants tended to acknowledge their illness when family support existed.

Though there are distinctions between the participants’ perception of their health and how family members perceive their health, different kinds of support and care from family members led to participants perceiving their health as being healthy. But family members perceived them as being ill, and limited their parenting and mobility due to the higher likelihood of accidents and hospital expenditures as a result. This led the majority of the participants to become economically dependent for their health condition, especially for buying medicines, on their children. According to P(6).

*“Now I am feeling a little better, so the medical expenditures were borne by my son. He keeps asking me what medicines I need and procures them for me. The medicines that I don’t get nearby, I ask him to get them for me from where it’s available, like the thyroid medicine which is not available nearby. It costs Rs 500 per month for my medicines*”.

Even though the majority of the participants received economic support from their children, few participants continued to work (explained in detail in the work and occupation section below). These economic and non-economic engagements led participants to perceive their health as healthy as they were able to take care of their own household expenditures.

Family caregiving was also reflected in whether they were accompanied when seeking care. Few participants were accompanied by their children when going to the doctor, P(5) stated, *“My elder daughter takes care of me. She takes me to the doctor for my check-ups and also gets me the necessary medicines*”. In addition to medicines, participants also said that their family maintenance is fully supported by their children P(8) said,

*“I have two children who will take care of me and our family economically. We have got them married and we are a bit peaceful now. If there’s tension, we sit as a family and talk and that helps relieve it. I can’t earn at this age and my husband’s eyesight also is deteriorating*”.

Few participants said if their children are not living with them, then they stay with their grandchildren P(14) elaborated,

*“Our son got married and lives elsewhere. Both my daughter and daughter-in-law take good care of us. Sometimes my BP goes high; I had to be admitted to the hospital two or three times. I still have trouble so I got one of my granddaughters, who is studying in the 9th standard, to stay with me and help me. She doesn’t want to study beyond SSLC, so she stays with me and studies Arabi Quran. I help her study the Quran and she helps me*”.

The presence of grandchildren provides emotional support along with support in household chores as P(7) articulates “*She is a child but gives me a lot of moral support. She keeps saying don’t stress yourself, Allah will take care of everything. That makes me feel better*”. Therefore, family support and caregiving in the form of belonging, care-seeking and economic support towards medicines and household expenditure have allowed participants, to an extent, to deal with and manage their conditions.

On the other hand, other participants also expressed receiving no or very limited support and care from their children. This lack of psychological and economic support played a role in enhancing the already existing feeling of illness P(10) articulated *“No one takes care of me. If my son doesn’t take care of me, who will take care?*”

### Health perceptions through work and occupation

Participants articulated their perception of being healthy or being ill based on their ability to perform work (including both paid and unpaid) before and after their diagnosis. Although the perception of being healthy or ill was articulated in terms of the ability to perform work, we also witness that the decision to continue occupations depended on one’s perception of their own health.

Among the participants interviewed, majority of them were previously working in paid jobs. All the women participants, except for one, were engaged in unpaid domestic work in addition to their occupation, while the same was true for just one man, who had to raise his children after the death of his wife. All the participants with an occupation were engaged in informal work or enterprises such as hospital housekeeping staff, electrician, beedi making, incense stick making, basket weaving, household domestic labour, tailor, contract bus driver, hat seller, owner of a small kirana store shop, barber in men’s saloon. One-woman participant was engaged solely in unpaid domestic work and considered herself as a homemaker. Most participants were only able to make their ends meet with the help of income generated from their occupation which ranged from INR 2000–3000/- per month in the case of household help to INR 10,000/- for the contract bus driver.

With the onset of the chronic disease, except for two participants, the nature of their work transformed. The majority of them have left their occupation. The commonly cited reason was “*susst*” (tiredness) and “*kamjori*” (weakness), which indicate that they perceived their health as being ill after their diagnosis. The contract bus driver, who took a voluntary retirement from the service, when asked about leaving work, said:

I retired because of diabetes. I used to physically feel weak and I was afraid that if some unfortunate incident took place, I would lose the retirement perks of my job and also, I did not want to risk the people’s lives. So, I took early retirement from the job P(8).

When asked how their chronic conditions impacted the ability to work, most participants equated their occupation with their life. This was one of the most common responses irrespective of gender. One participant mentioned,

With diabetes, life changes. Earlier I used to work for 8 h and not get tired. Now, with diabetes, even if I work for 5 h I get tired and my body needs to rest P(9).

When asked further to describe the bodily reaction, he continued,

I feel some kind of rush from inside and my body muscles stop functioning. I sweat so much that the cloth I use to wipe my sweat becomes very wet P(16).

A similar response was received from another participant who had both diabetes and hypertension,

After being diagnosed with diabetes, I started feeling weakness in my hands and legs. The amount of work I used to do before, I cannot do it now. Even hypertension affects me. If I go into the sun, I face difficulty. If I feel any stress my BP shoots up. Earlier this was not the case, I used to work for many hours P(12).

In the case of their own household work, except in one case where the participant lost his wife, it was only women, either daughters, daughter-in-law’s or granddaughters- who were engaging in domestic labour along with their occupation. In the case of the one male participant who was diagnosed with diabetes and lost his wife, responsibility of household work was transferred to his newly married daughter-in-law.

After the onset of the disease, in the majority of the women’s participant’s homes, domestic labour was either fully or partially passed on to their daughters and granddaughters.

There was no one to perform household chores. My elder son got married and now stays separately. My younger son stays with me and I used to take care of him by cooking and helping him with his daily needs. Performing these household chores used to make my BP increase and I was admitted in the hospital a couple of times. After these incidents, I got one of my granddaughters (daughter’s child) to stay with me and help me with household chores P(7).

Similarly, another participant shares how her granddaughter helps her as follows;

This is my elder daughter’s child. Because of my health condition she stays with me and helps me with household chores like cooking and going to college. She is in her second year of college now P(5).

It was also noticed in a few cases that children discouraged the diagnosed parent from continuing their previous occupation and instead helped them set up a small enterprise, mostly local kirana stores, adjoining their homes so that they could continue to earn with a reduced workload.

While in almost all cases it was the children who took over family responsibilities, in the case of one participant, it was his wife who started working when her husband was diagnosed with hypertension and got partially paralysed. The participant, when asked if he was okay with his wife working outside home, he responded, “*What other option do we have?*‘’ He also went on to add, “*What is the necessity for my wife to go and work outside if I am able*?”

From the above narratives it can be understood that the participants’ perceptions of their health depend on their ability to work, and are expressed through words such as *susst* and *kamzori*. Likewise, one’s perception of health also determined one’s decision making process of continuing occupation.

### Health perceptions through lifestyle

The perception of health is also shaped by changes in the participants’ lifestyles after the diagnosis of diabetes and hypertension. Participants highlighted how the diagnosis and adherence to the treatment for diabetes and/or hypertension brought changes in their lifestyle. P(3) stated, *“I used to eat everything before I was diagnosed with diabetes and BP. Even now I eat sweets, but it is limited. Because of BP, I reduced my salt intake through food*”. Another participant P(7) added.

“*I reduced consumption of meat, sweets and everything. Some people consume everything even after diagnosis, but I don’t behave that way. I always have my child on my mind. If something happens to me, he will suffer a lot mentally and economically because he has to admit me in the hospital and take care of me*”.

Participants also remarked on the lifestyle changes due to medicine intake after diagnosis. P(10) articulated, *“Nothing changed actually and everything’s normal. Earlier I didn’t take medicine and now I have to—that’s the only change. Earlier there was no BP so I didn’t have to take tablet, now I have to take it every day, that makes me a little weak*”. Changes in lifestyle by daily intake of medicines led participants perceive their health as being ill.

Participants also highlighted the difficulties in maintaining behaviour changes in their drinking habits, physical activity, and food habits. Though participants did not elaborate much on tobacco consumption and drinking habits, overall there was a reduction in tobacco and alcohol consumption after the diagnosis. Drinking was found mostly among male participants, and was partially or fully stopped after the diagnosis. P(11) articulated “*Now I am under treatment, so I don’t consume alcohol*” P(15) added “*If it’s on my mind to drink, then I drink or else I don’t”.*

In the case of physical activity, a majority of the participants expressed that they do not have a fixed time allocated for walking. Participants connected physical activity with their household chores, occupation and physical discomfort. P(1), who runs a kirana shop, remarked, *“I get to walk here only a lot. At 6:30 in the morning I walk to get milk. Several times in the day I walk to get other things such as groceries and vegetables. If it’s a little far, I stop for short rests in between. I generally don’t take the auto rickshaw, bus or other motor vehicles unless it’s too far”*. The interface of being healthy and ill is reflected through the participants weakness during walking and their willingness towards walking.

Another participant P(8) expressed relief from their *taqleef* (discomfort) due to active walking which led the participants perceive their health as being healthy, *“It’s better if I walk, otherwise I feel pain. If I keep walking then there’s no issue but if I sit for long hours then I feel like my legs are getting stuck in one place*”. On the contrary, walking didn’t give relief to P(7) and continued to perceive health as being ill, who said, *“I walk to work, so it covers my exercise needs. Earlier, I used to go for a walk irrespective of my work but then had to stop because of pain in my legs and back*”. Similarly, P(15) added, *“I used to go for a walk in the morning and then stopped because it used to cause pain in my legs and breathing issues. If I climb down the stairs it gets difficult to breathe”.*

Food habits also changed or remained unchanged after diagnosis for participants due to the adherence or non-adherence to the NCD-appropriate diet. It was difficult for few participants to strictly follow the doctor’s advice on their diet due to various reasons. One such reason was changes in taste due to continuous intake of medicines, P(19) articulated as *“Because of medicines, my taste gets spoiled so I need something tasty. But then, I get scared that my BP may get high because of eating such food”.* Here the eating habit, i.e., perceiving heathy while non-adhering to the NCD-appropriate diet and being ill while adhering to the diet, by the participants clearly reflect the interfaces of being healthy and being ill. Further, no changes in food preparation at household level led to non-adherence to the appropriate diet, but most of the participants said that they avoid outside food, P(7) articulated

*“We prepare food in the home itself, and don’t consume outside food. We get groceries and vegetables and prepare it at home. Outside food does not sit well with me”.* P(11) said, in case of food preparation, “*Even though I have BP, there’s no separate food prepared for me with less salt. There’s only one type of food prepared for everyone that has normal levels of spice and salt. If my health gets bad, then rice starch is made for me. Once I get better I eat “normal” food along with other family members”.* Here too the perception of being healthy is reflected through eating normal food i.e., non-adhering to the NCD-appropriate diet.

A few participants were habituated with their prescribed diet, P(18) articulated

“*I eat roti mostly and I am used to it. If there’s no roti then I eat a little rice in the afternoon and that’s it. After being diagnosed with diabetes, I started avoiding rice and eating wheat roti*”.

A few participants also did not care about sticking to a diet at all due to the lack of sufficient food. One participant, P(9) said, *“I eat whatever is available. There’s no access to better facilities. Whatever I get to eat, I believe it’s because of Allah”*. Therefore, it is clear that adhering to an NCD-appropriate diet is given very less importance while adhering to treatment of diabetes and hypertension. Diet restriction is also difficult to maintain for many participants given their difficulties in eating food on time while at work and occupation, and further inadequate quantity of food and types of food in NCD-appropriate diet is inefficient to maintain a productive lifestyle.

## Discussion

Perception of health has been studied commonly in the context of communicable diseases [26, 28, 29], maternal [11, 12, 19, 30, 31] and child health [1, 2]. In the context of NCD’s, there is a tradition of examining behaviours and perceptions through various lens such as self-perceived health [6, 7], self-construction of illness [10] social construction of illness [8, 13, 18, 34] health perceptions [5, 20, 23, 27]. However, in the context of NCDs and associated chronic conditions, there is a serious dearth of research on low-income population in developing countries, which this study has attempted to address.

As discussed in the results section, the episodic nature of being healthy and being ill as experienced by most of the participants while undergoing treatment is a clear indication of the physiological imbalances through the controlled and uncontrolled state of diabetes and/or hypertension. This is strongly determined by the factors such as presence of family support and caregiving, changes in work and occupation, changes in lifestyle, psychological stress from being diagnosed and other familial problems. The diagnosis itself and intake of medicines made few participants perceive their health as being ill and acknowledge their illness. The perception of being healthy or being ill is captured mostly through these factors in a bi-directional manner i.e. the effect of social factors on perceptions of health and vice versa. Kleinman [16], and Mendenhall and colleagues [21] also point to similar findings through their studies, that individual experiences of health were influenced by a variety of cultural values and social norms that were collectively reinforced by the caregivers, healthcare workers and systems.

The episodic nature of being healthy and being ill is experienced by all the participants throughout their illness, even after recognising the severity of their symptoms before the diagnosis of diabetes or hypertension. Physical discomfort made participants perceive their health as being ill, but it is unlikely that the participants considered them as symptoms, especially since there was a significant delay in their diagnosis and the initiation of treatment before the diagnosis. After the diagnosis, the majority of the participants were much aware of the connections between symptoms involving discomfort and the controlled and uncontrolled state of diabetes and hypertension, for instance, that headaches lead to increased hypertension and dental problems lead to increased sugar levels. Physical discomfort is described by the participants as body pain, leg pain, headache, difficulty in walking, breathing problem, and tiredness, which they have experienced episodically or on a daily basis. But in most cases, this was ignored due to economic and non-economic demands. These demands increase psychological stress, and in turn lead to perceived health as being ill. But when social support from family members, especially children, was present, there was a temporary reduction in stress, which made participants perceive their health as healthy.

This study found that family support and caregiving have a strong impact on how participants perceive their health. Stress was found to be a common problem among all the participants and was reported as the main reason for getting a diagnosis with diabetes and hypertension. A few participants, however, who lost their spouse/children, had family members with illnesses/disabilities or were not supported by their children, experienced high levels of stress compared to the other participants. They, participants, described their situation as their *taqdeer* (fate) and with a sense of acceptance. To make up for the absence of family members, these participants either bring a granddaughter or a child (usually female) from their siblings to live with them. Alongside school, these children provide caregiving support, mainly by giving injections and psychological support, helping with household chores, and reminding participants about their medications. This kind of support led participants to feel better about their health. Further, there was a clear difference found between how participants perceive their health and how their family members perceive it. This study found that children and extended family members perceive the participants’ health as being ill and often attempted to limit the participants’ parenting activities and mobility to avoid possible future healthcare expenditures. This led to the participants discontinuing or switching their occupation and becoming fully or partially dependent on their children for care-seeking and purchasing medicines. But participants accepted this as care from their children, which led them to perceive their health as healthy. Besides, participants also perceived their health as being ill while connected to their inability to work. The diagnosis of diabetes and hypertension had a significant impact on their productivity, in both economic and non-economic engagements. But few participants continued to work (mostly irregular work) for income, that does not goes beyond their bodily limits i.e. uptake of activity based on their state of physical discomfort or normalizing their physical discomfort, since earning an income is the top priority to manage daily necessities including out-of-pocket health related expenditures for them and their family. This is an indication of them perceiving their health as healthy.

A study conducted by Mendenhall and colleagues [22] in Delhi revealed how diabetes is normalised in everyday life and not considered an illness. The majority of our study participants considered diabetes and hypertension as illnesses given their impact on their lives and lifestyle, like having to switch/leave their occupation, consume medicines, and adhere to lifestyle practices while undergoing treatment. But these behaviours fluctuated depending on whether the participants perceived their health as healthy or ill throughout the illness experience. Therefore, this study acknowledges it as normalizing health rather than normalizing an illness, i.e., normalizing the symptoms and behavioural actions and not the diabetes and/or hypertension. Their lifestyle changes, especially walking and following the appropriate diet, are influenced by socio-economic factors. This study found that variations in the participants’ perception of health is very evident from their behavioural practices. The participants who had left their occupation or switched to another occupation where mobility is restricted expressed that they do not have a dedicated time allocated for walking. Perceiving health as healthy and ill was often experienced through the ups and downs of physical discomfort, which led them to not walk, sit/rest while walking, walk slowly, and walk inefficiently and irregularly. Though few participants expressed that their physical discomfort reduces while they walk, there was no connection found between walking and body weight except one participant who articulated that obesity had led to reduced or no walking due to severe physical discomfort in the lower limb. Similarly, adhering to the diet was also followed by a majority of the participants but only while experiencing moderate or severe physical discomfort (which many times led to visiting a doctor). Thus, the perception of health fluctuates based on recognising physical discomfort. Overall, it was found from our analysis that participants perceived their health as being ill but the behavioural actions are mostly confined to being healthy except for very few cases. There was also a strong relationship found between socio-economic factors and these behavioural actions which streamline the behavioural actions to fluctuate between being healthy and being ill.

In conclusion, the decision-making for health through health perception began even before the diagnosis of diabetes and hypertension and continued throughout the illness experience. The participants’ perception of their health as healthy or ill strongly influenced this decision-making. This study found many interfaces of being healthy and being ill in an episodic manner while experiencing an illness. Therefore, it recommends considering health and illness as two different entities while researching chronic conditions. It is important for the health system to understand and fix these episodes, which often lead to switching between controlled and uncontrolled states of diabetes and hypertension. To fix the episodes of being healthy and being ill, it is important to improve the social, economic, behavioural and psychological factors. The interplay between these factors has socialized health perception and various related practices from the individual to the community level. Therefore, the health system needs to re-strategize its focus from individual to community level interventions to address the determinants of health and NCD risk factors by strengthening the NCD prevention approach. Further, this study also prompts rethinking how the health system can be strengthened towards addressing and considering the social, economic, behavioural, and psychological factors in improving an individual’s health outcome. Why doesn’t the health system speak in the same way as the people speak of chronic illness?

## Data Availability

Data sharing is not applicable to this article as no quantitative datasets were generated or analysed during the current study. The qualitative data is included to an extent in the result section of this manuscript.
